# Implementation and evaluation of the ‘Transgender Education for Affirmative and Competent HIV and Healthcare (TEACHH)’ provider education pilot

**DOI:** 10.1186/s12909-021-02991-3

**Published:** 2021-11-04

**Authors:** Ashley Lacombe-Duncan, Carmen H. Logie, Yasmeen Persad, Gabrielle Leblanc, Kelendria Nation, Hannah Kia, Ayden I. Scheim, Tara Lyons, Chavisa Horemans, Ronke Olawale, Mona Loutfy

**Affiliations:** 1 School of Social Work, University of Michigan, 1080 South University Avenue, Ann Arbor, MI 48109-1106 USA; 2 Center for Sexuality and Health Disparities, University of Michigan, 400 North Ingalls Street, Ann Arbor, MI 48109-5482 USA; 3 Women’s College Research Institute, Women’s College Hospital, 76 Grenville Street, Toronto, Ontario M5G 1N8 Canada; 4 Factor-Inwentash Faculty of Social Work, University of Toronto, 246 Bloor Street West, Toronto, M5S 1V4 Canada; 5 Center for Gender & Sexual Health Equity (CGSHE), 1190 Hornby Street, Vancouver, V6Z 2K5 Canada; 6 Action Santé Travesti(e)s & Transsexuel(le)s du Québec (ASTT(E)Q), 1300 Sanguinet, Montréal, H2X 3E7 Canada; 7 Prism Education Series, Vancouver Coastal Health, 1128 Hornby Street, Vancouver, V6Z 2L4 Canada; 8 School of Social Work, University of British Columbia, 2080 West Mall, Vancouver, V6T 1Z2 Canada; 9 Epidemiology and Biostatistics, Drexel Dornsife School of Public Health, Drexel University, 3215 Market Street, Philadelphia, PA 19104 USA; 10 Department of Criminology, Kwantlen Polytechnic University, 12666 72 Avenue, Surrey, V3W 2M8 Canada; 11 CIHR Canadian HIV Trials Network, 588-1081 Burrard Street, Vancouver, V6Z 1Y6 Canada; 12 Department of Medicine, University of Toronto, 1 King’s College Circle, Toronto, M5S 1A8 Canada

**Keywords:** Transgender, Stigma, Health care, Provider, Social services, HIV prevention, HIV treatment, Barriers to care

## Abstract

**Background:**

Transgender (trans) women face constrained access to gender-affirming HIV prevention and care. This is fueled in part by the convergence of limited trans knowledge and competency with anti-trans and HIV-related stigmas among social and healthcare providers. To advance gender-affirming HIV service delivery we implemented and evaluated ‘*Transgender Education for Affirmative and Competent HIV and Healthcare (TEACHH)’*. This theoretically-informed community-developed intervention aimed to increase providers’ gender-affirming HIV prevention and care knowledge and competency and reduce negative attitudes and biases among providers towards trans women living with and/or affected by HIV.

**Methods:**

Healthcare and social service providers and providers in-training (e.g., physicians, nurses, social workers) working with trans women living with and/or affected by HIV (*n* = 78) participated in a non-randomized multi-site pilot study evaluating TEACHH with a pre-post-test design. Pre- and post-intervention surveys assessed participant characteristics, intervention feasibility (e.g., workshop completion rate) and acceptability (e.g., willingness to attend another training). Paired sample t-tests were conducted to assess pre-post intervention differences in perceived competency, attitudes/biases, and knowledge to provide gender-affirming HIV care to trans women living with HIV and trans persons.

**Results:**

The intervention was feasible (100% workshop completion) and acceptable (91.9% indicated interest in future gender-affirming HIV care trainings). Post-intervention scores indicated significant improvement in: 1) knowledge, attitudes/biases and perceived competency in gender-affirming HIV care (score mean difference (MD) 8.49 (95% CI of MD: 6.12–10.86, *p* < 0.001, possible score range: 16–96), and 2) knowledge, attitudes/biases and perceived competency in gender-affirming healthcare (MD = 3.21; 95% CI of MD: 1.90–4.90, *p* < 0.001, possible score range: 9–63). Greater change in outcome measures from pre- to post-intervention was experienced by those with fewer trans and transfeminine clients served in the past year, in indirect service roles, and having received less prior training.

**Conclusions:**

This brief healthcare and social service provider intervention showed promise in improving gender-affirming provider knowledge, perceived competency, and attitudes/biases, particularly among those with less trans and HIV experience. Scale-up of TEACHH may increase access to gender-affirming health services and HIV prevention and care, increase healthcare access, and reduce HIV disparities among trans women.

**Trial registration:**

ClinicalTrials.gov (NCT04096053).

## Background

Barriers to gender-affirming HIV prevention and care for transgender (trans) women – a diverse group of people assigned male at birth who typically identify as women, trans women, and/or transfeminine – are an enduring health equity issue for those living with and affected by HIV. These barriers contribute to disproportionately high rates of HIV in comparison to cisgender (cis) adults (those whose gender identity and sex assigned at birth are congruent) [1]. A recent meta-analysis identified an HIV prevalence of 14.11% (95% confidence interval [CI]: 8.70–22.22%) among trans women across 13 United States (U.S.) studies [1], whereas the U.S. national HIV prevalence is approximately 0.36% [2].

Access to HIV prevention, treatment, and support are critical to realizing optimal health and wellbeing among trans women [3, 4]. Thus, a key issue is that compared to cis persons, trans women have lower access to HIV testing [5]. Also, trans women living with HIV are less likely to be retained in care [6, 7], take antiretroviral therapy (ART) [8], adhere to ART [9–12], and to be virally supressed [7, 12, 13] compared to cis people living with HIV. Gender inequities with respect to HIV care access also intersect with racial disparities. For example, compared to Black cis women, Black trans women are less likely to be retained in care and virally suppressed [7]. Pre-exposure prophylaxis (PrEP), an HIV prevention technology that involves the use of ART, generally in the form of a once-daily pill, can substantially (> 95%) reduce HIV acquisition risk [14]. Yet gender-based disparities also exist in PrEP uptake, such that trans women are less likely to be aware of PrEP, to have discussed PrEP with a healthcare provider, and to use PrEP, compared to cis men who have sex with men (MSM) [15].

### Provider-level barriers to HIV prevention and care affecting trans women

Well-documented barriers to trans women’s access to HIV prevention and care are stigmas related to trans identity, HIV, and other characteristics, perpetuated by healthcare and social service providers [16–22]. For example, results of a mixed-methods study of HIV care access among trans women living with HIV showed that trans-related stigma was negatively associated with having accessed HIV care in the year preceding the survey, and both trans-related stigma and HIV-related stigma were associated with lower likelihood of ART use [21]. Such stigmas ranged from subtle microaggressions to outright denial of care [21]. Other qualitative work [22] similarly identified how anticipated and enacted trans-related and HIV-related stigma in healthcare among health professionals, along with a lack of provider knowledge of HIV care needs for trans women, prevented trans women from accessing HIV testing and competent care. An additional related barrier is the lack of healthcare provider knowledge regarding trans healthcare and a lack of competency to provide care that is gender-affirming to trans patients.

Numerous studies have documented a continued gap in content specific to lesbian, gay, bisexual, trans, and queer (LGBTQ) content in healthcare education [23–28]. Providing gender-affirming care is more than the absence of anti-trans stigma and encompasses more than providing medical transition-related care. A provider may be considered to be competently providing *gender-affirming care* when they: 1) have foundational knowledge about issues associated with trans people in addition to understanding appropriate terminology; 2) reconcile personal beliefs, biases and attitudes towards trans people with their professional role; 3) actively work towards creating an inclusive healthcare environment; and 4) support patients in defining their gender and their relationship with gender [29, 30]. While HIV retention and care intervention studies have focused largely on the individual-level (e.g., peer networking) [31], less attention has been paid to shifting the organizational contexts through educating health and social service providers in gender-affirming HIV prevention and care [32].

### Provider education workshops

A systematic review of trainings to reduce LGBTQ-related bias among medical, nursing, and dental students and providers (*n* = 13 studies) reported that these interventions were effective at increasing knowledge of LGBTQ healthcare issues, comfort level towards working with LGBTQ patients, and tolerant attitudes towards LGBTQ patients [33]. However, provider education interventions have predominantly focused on LGBTQ people broadly, as opposed to trans people more specifically [33]. This constitutes a significant gap and conflates gender and sexual orientation. For example, a recent study conducted with 252 medical students showed that confidence discussing sexual orientation with a patient increased with year of study but confidence discussing patient gender identity did not [34].

That said, preliminary studies suggest that educational workshops are a promising approach to increase providers’ knowledge and competency to provide gender-affirming care [35, 36]. Lelutiu-Weinberger et al. [35] evaluated the use of three, 2-hour workshops focused on improving competency to provide gender-affirming clinical care and reducing trans-related stigma among 35 healthcare providers working in New York City including those in direct (e.g., physicians) and indirect (e.g., administrative staff) roles. Study findings showed decreased negative attitudes, and increased staff awareness of transphobic practices, willingness to serve trans persons, and representation of general LGBT-related visuals at the clinic’s reception area [35]. Similarly, Allison et al. [36] demonstrated that these changes could also be seen with a brief single-session intervention, describing significant improvements in knowledge, interpersonal comfort, and sex and gender beliefs among 58 students who took part in a 2-hour interprofessional education workshop focused on gender-affirming care. Notably, this study also took place in the U.S. There is a dearth of provider intervention literature emerging from Canada, which, despite having had federal human rights protections for trans persons since 2017, still has documented disparities in healthcare access among trans people [37]. For examples, findings from a survey conducted with 2, 873 trans and non-binary people across Canada in 2019 found that 45% of participants had unmet healthcare needs in the past year, and 12% had avoided the emergency room in the past year, despite needing care [37]. These findings suggest additional interventions may be needed to address healthcare barriers for trans people in Canada.

Limited literature exists regarding provider-level interventions to increase gender-affirming care competency, despite that community-led initiatives, such as the U.S.-based Transgender Training Institute and the Canadian-based The 519 have been undertaking these efforts for years if not decades [38, 39]. However, there is much literature showing success of provider-level interventions in reducing HIV-related stigma [40–44]. For example, Batey et al. [41] assessed the feasibility and acceptability of the Finding Respect and Ending Stigma around HIV (FRESH) intervention, a theoretically-informed 1.5 day workshop aiming to sensitize both people living with HIV (PLWH) and healthcare providers to HIV-related stigma and to encourage collaborative development of further provider HIV stigma reduction intervention efforts. Findings of the pilot test with 17 healthcare providers and 19 PLWH found positive and statistically significant trends towards increased awareness of stigma in healthcare settings among providers [41].

### Gaps in the literature

A growing body of research recommends service provider training as a strategy to increase HIV prevention and care access for trans women living with and/or affected by HIV [21, 45]. Indeed, access to gender-affirming healthcare [46, 47], including non-judgmental and gender-affirming clinic environments [48, 49], can promote access to HIV prevention and care among trans women living with and/or affected by HIV. However, despite these recommendations, few studies have described or evaluated provider training to improve gender-affirming HIV care competency among providers caring for trans women.

Another significant gap in the aforementioned provider education interventions is that they address only one type of stigma (e.g., anti-trans OR HIV stigma) [29, 30, 40–44]. Intersectionality, a critical social theory rooted in Black feminism [50, 51], addresses the need to consider multiple sources of oppression that produce health inequity [52]. This theoretical and applied framework exists both as a response and a challenge to ‘single axis’ approaches that focus on oppression based on singular dimensions of social location (e.g., gender identity) [53]. As trans women experience intersecting stigmas when accessing HIV prevention and care [21, 22, 54–56], intersectionality is highly applicable for informing research involving this group. For example, one experimental study assessed HIV-related stigma behaviors among medical students during three standardized patient interactions with patients experiencing intersecting stigma based on substance use, sexual orientation, and/or sexual practices (e.g., condomless sex), relative to a control condition [56]. Study findings showed a higher number of stigma behaviors manifested towards all experimental conditions compared to the control condition, with the highest levels of HIV-related stigma directed towards sexual minority men [56]. Together, these studies signal the importance of developing a provider education intervention focused on improving gender-affirming practice by reducing HIV-related and trans-relatedstigma.

### The current study

The current study details the pilot testing of ‘*Transgender Education for Affirmative and Competent HIV and Healthcare (TEACHH)’*, a theoretically-informed provider education intervention [57] for healthcare and social service providers, along with providers-in-training. The ultimate goal of TEACHH was to increase providers’ ability to provide gender-affirming HIV prevention and care to trans women by increasing their gender-affirming HIV knowledge and perceived competency, while also reducing negative attitudes and biases towards trans women.

## Methods

### Study design and participants

We utilized a non-randomized multi-site pilot study with pre- and post-test design to implement and evaluate the TEACHH intervention with healthcare and social service providers (e.g., physicians, nurses, social workers) and providers in-training working with trans women living with and/or affected by HIV from September to December 2019. In Toronto, the research team identified relevant organizations (e.g., AIDS Service Organizations), and elicited support from organizational leadership to host TEACHH within each organization. Student participants (*n* = 4) were embedded as learners within these organizations. We were intentionally inclusive during this pilot stage, encouraging those with multiple roles within these organizations, including administrative and managerial, to engage in the training. In Vancouver, the team recruited participants through social networks (e.g., Facebook), in addition to reaching out to specific trans and/or HIV care providers via email, then held two workshops in a central location with providers from multiple organizations.

All participants provided written informed consent prior to participating in the intervention. All study procedures were approved by the University of Toronto Research Ethics Board and registered with ClinicalTrials.gov (NCT04096053).

### TEACHH intervention structure and content

The community-based process of TEACHH intervention development is detailed elsewhere in a study protocol [57]. In brief, our community-based approach involved reciprocity, capacity-building, and knowledge development with, by, and for trans communities [58]. Team members have long histories of community-based trans education in their respective cities, thus TEACHH was adapted initially from ‘Trans 101’ trainings offered in Montreal, Toronto, and Vancouver, Canada, by these team members. We utilized four of Card’s [59] seven steps of adapting interventions combined with four steps from the ADAPT-ITT model for adapting evidence-based HIV interventions to develop the final version of TEACHH [60]. TEACHH was underpinned by the Information-Motivation-Behavioral Skills Model (IMB) [61], a health behavior change theory previously applied in an LGBT provider cultural competency intervention [62] that accounts for how knowledge, motivation, and skills interact to influence provider behavior. Findings from focus groups with 26 trans women and 10 service providers further informed intervention development [22]. Specifically, TEACHH sought to address providers’ gender-affirming HIV care knowledge, perceived competency to provide gender-affirming HIV care, and attitudes/biases towards trans women living with HIV. TEACHH was delivered by trans women in a 3-h session. Contents of the TEACHH intervention mapped to intervention objectives can be seen in Table 1.

**Table 1:** TEACHH Intervention Training Topics Mapped to Intervention Objectives

		Intervention Objectives Met		
#	Topic	Increase Knowledge	Reduce Attitudes/Biases	Increase Perceived Competency
1	Human rights for and intersecting stigmas affecting trans women	*✓*	*✓*	
2	Affirming words to discuss gender identity and expression	*✓*		*✓*
3	Basic understanding of trans healthcare (e.g., hormone therapy), HIV prevention (e.g., pre-exposure prophylaxis), and HIV treatment (e.g., ART), and how these affect trans women living with HIV	*✓*		
4	HIV-specific social movements (e.g., U=U or undetectable = untransmittable)^a^	*✓*	*✓*	
5	HIV laws and policies (e.g., criminalization of HIV non-disclosure)	*✓*	*✓*	
6	Strategies for enhancing gender-affirming service provision with individuals and at the organizational level	*✓*		*✓*

^a^Note. U=U is a movement promoting the evidence-base showing that PLWH who receive ART and have achieved and maintained an undetectable viral load cannot sexually transmit the virus to others [63])

At the end of the intervention, participants completed a case study describing a potential client’s intersectional experiences (e.g., trans women who are immigrants/newcomers), to apply what they learned to practice (application of knowledge, interpersonal strategies, and organizational strategies) [57].

### Evaluation components

We administered a pre-intervention questionnaire that collected sociodemographic characteristics and employment and past training experiences of participants, as well as two measures assessing provider perceived competency, attitudes/biases, and knowledge. A follow-up questionnaire was then administered immediately post-intervention that assessed acceptability and, again, perceived competency, attitudes/biases, and knowledge.

#### Sociodemographic characteristics

We asked participants about trans community membership (yes/no), sexual orientation (open-ended then categorized as heterosexual, gay, lesbian, bisexual, queer, other sexual orientation, or more than one sexual orientation), race/ethnicity (open-ended then categorized as White, Black/African/Caribbean, Latina/o/x, Asian, Southeast Asian, multiple races or ethnicities, or person of color (not specified), and age (continuous).

#### Employment and past training experiences

We collected participant role, categorized as social service, medical or other allied healthcare provider (e.g., physiotherapy), administrator at the management level, administrative assistant, student, research, multiple roles. We also asked about the number of trans clients served in the past year, number of those trans clients who identified as trans women or transfeminine, and number of those trans women clients who were living with HIV, hours of past training about trans identities/communities (none, < 1 hour, 1–3 hours, > 3 hours), hours of training specific to the needs/experiences of people living with HIV (none, < 1 hour, 1–3 hours, > 3 hours), and whether or not participants had ever received training specific to needs/experiences of trans women living with HIV (yes, no). Finally, we asked participants to qualitatively describe their past training experiences.

#### Intervention feasibility and acceptability

As a pilot study, our primary outcomes were feasibility and acceptability [64]. Feasibility was measured through workshop completion rate, research consent rate, pre- and post-intervention measures’ completion rates, and average length to complete pre- and post-intervention questionnaires.

Acceptability questions included participant reasons for attending the workshop, overall satisfaction with the workshop, and willingness to attend another workshop on trans women and HIV. We also elicited open-ended feedback on TEACHH (most beneficial aspects, missing information, overall thoughts).

#### Perceived competency, attitudes/biases, and knowledge

We examined secondary outcomes of perceived competency, attitudes/biases, and knowledge needed to provide gender-affirming HIV care to trans women living with and/or affected by HIV. Given the lack of measures specific to assessing these areas at the intersection of trans identity and HIV, we created one measure. We also adapted a measure to capture general (non-HIV-specific) knowledge, attitudes/biases, and perceived competency for gender-affirming healthcare. Measures are available in our published study protocol [57].

The first measure assessing Perceived Competency, Attitudes/biases, and Knowledge to Provide Gender-Affirming HIV Care (Gender-affirming HIV Care), was a 16-item measure combining three items from Nyblade et al.’s [65] brief, standardized tool for measuring HIV-related stigma among health facility staff with 13 newly created items that assess attitudes/biases (3 items, e.g., “If I had a choice, I would prefer not to provide services to trans women living with HIV”), perceived competency (7 items, e.g., “I am comfortable prescribing or referring patients to a physician who will prescribe both feminizing hormones and PrEP”), and knowledge needed to provide gender affirming HIV care (6 items, e.g., “I am knowledgeable about the barriers trans women experience when accessing care/treatment for HIV”). Response options to each item ranged from Strongly Disagree = 1 to Strongly Agree = 6, thus with a possible score range of 16 to 96. With three items reverse coded, a total score indicated higher perceived competency, more positive attitudes, and higher knowledge (Cronbach’s α = 0.90 [pre-test score]).

The second measure, assessing Perceived Competency, Attitudes/Biases, and Knowledge to Provide Gender-Affirming Healthcare (Gender-affirming Healthcare), was a 9-item measure closely adapted from the trans-specific questions from Bidell’s [66] Lesbian, Gay, Bisexual, and Transgender Development of Clinical Skills Scale (LGBT-DOCSS), an interdisciplinary self-assessment for health providers (e.g., “I feel competent to assess a person who is transgender in a therapeutic setting”; “Allowing children and teenagers who believe they are transgender to take hormones is wrong”). This measure was not specific to trans women living with HIV but assessed the three areas more broadly in relation to trans people. Response options to each item ranged from Strongly Disagree = 1 to Strongly Agree = 7, thus with a possible score range of 9 to 63. With four items reverse coded, a higher score indicated higher perceived competency, positive attitudes, and knowledge (Cronbach’s α = 0.71 [pre-test]). The study team chose to including both an intersectional measure (trans identity and HIV experience) and a trans-specific measure, to determine if we could address intersectional gaps or trans-specific gaps alone.

### Data analysis

All variables were summarized as frequencies and proportions (for categorical variables) and means and standard deviations or medians and interquartile ranges where appropriate (for continuous variables). Open-ended questionnaire responses were analyzed using conventional qualitative content analysis methods and summarized thematically [67]. Specifically, one of the authors (RO) open-coded the open-ended questionnaire responses, then met with a second author (ALD) to review the open-ended codes and to create higher-level themes within the overarching areas of most beneficial aspects (e.g., content of training), missing information (e.g., more in-depth medical content), and overall thoughts (e.g., general positive feedback). To assess pre- and post-intervention differences in two measures, we used paired sample t-tests. Cohen’s d (effect size) was also reported, with 0.2, 0.5, and 0.8 considered cut-offs for small, moderate, and strong effect sizes, respectively [68]. Lastly, we conducted bivariate analyses to assess for potential associations between employment and past training of participant characteristics and pre- to post-intervention change score on both measures. To do so, we utilized Pearson’s correlation coefficient for continuous variables (e.g., number of trans clients served in the past year), and T-tests for categorical variables which were all dichotomized, including role (direct service provider [social service provider, medical provider, or other allied health provider] vs. indirect service provider [manager, administrative assistant, researcher]), hours of past training about trans identities/communities (none vs. any), hours of training specific to the needs/experiences of people living with HIV (none vs. any) and ever received training specific to needs/experiences of trans women living with HIV (yes vs. no). All analyses were conducted for those with complete data.

## Results

### Sociodemographic characteristics

Participants (*n* = 78 total; *n* = 60 Toronto; *n* = 18 Vancouver) were a mean age of 37.0 (standard deviation (SD): 11.0), predominantly cis (96.1%), heterosexual (66.2%), and people of color (75.0%), including Black, African, Caribbean, Latina/o/x, Asian, Southeast Asian, and multi-racial race/ethnicities (Table 2).

**Table 2:** Sociodemographic Characteristics of Participants (*n* = 78)

Characteristic	Mean (SD) or N (%)
City (*n* = 78)	
*Toronto*	60 (76.9)
*Vancouver*	18 (23.1)
Age (*n* = 74)	37.0 (11.0)
Gender Identity (*n* = 76)	
*Cisgender*	73 (96.1)
*Transgender or other gender diverse identity*	3 (3.9)
Sexual Orientation (*n* = 74)	
*Heterosexual*	49 (66.2)
*Gay*	10 (13.5)
*Lesbian*	2 (2.7)
*Bisexual*	1 (1.4)
*Queer*	6 (8.1)
*Other sexual orientation*	1 (1.4)
*More than 1 sexual orientation*	5 (6.8)
Race/ethnicity (*n* = 71)	
*White*	18 (25.0)
*Black, African, Caribbean*	27 (37.5)
*Latina/o/x*	5 (6.9)
*Asian*	7 (9.7)
*Southeast Asian*	9 (12.5)
*Multiple races or ethnicities*	5 (6.9)
*Person of color (not specified)*	1 (1.4)

### Employment and past training experiences

Participants held a range of direct service roles, mostly social service (56.4%) and medical or other allied health (21.8%) (Table 3). Participants also held a range of indirect roles including management (5.1%), administrative (non-management) (3.8%), and research (2.6%). Participants had worked with a median of 2 trans clients in the past year (Interquartile Range (IQR): 2, 10, Range = 0–50). Just over one-third (37.2%) reported receiving no past trans-specific training, whereas almost half (43.6%) reported having received more than 3 hours of such training. Among those who provided qualitative responses, participants had received past trans-specific training from a variety of sources, including a province-wide LGBTQ training organization (*n* = 9), local community-based organizations (*n* = 17), formal college/university-level courses (*n* = 4), workplace diversity training programs (*n* = 4), and self-education (*n* = 3).

**Table 3:** Employment and Past Training Experiences of Participants (*n* = 78)

Characteristic	Median (IQR), Range or N (%)
Role (*n* = 77)	
*Social Service Provider*	44 (56.4)
*Medical Provider or Other Allied Health (*e.g.*, Physiotherapy)*	17 (21.8)
*Administrator (Management)*	4 (5.1)
*Administrative Assistant*	3 (3.8)
*Student*	4 (5.1)
*Researcher*	2 (2.6)
*Multiple Roles*	3 (3.8)
Number of Trans Clients Served in Past Year	2 (2, 10), 0–50
Number of Transfeminine Clients Served in Past Year	1 (0, 1), 0–35
Number of Transfeminine Clients with HIV Served in Past Year	0 (0, 0), 0–20
Hours of Past Training About Trans Identities/Communities (*n* = 78)	
*None*	29 (37.2)
*< 1 hour*	4 (5.1)
*1–3 hours*	11 (14.1)
*> 3 hours*	34 (43.6)
Hours of Past Training About Needs/Experiences of Persons Living with HIV (*n* = 78)	
*None*	19 (24.4)
*< 1 hour*	3 (3.8)
*1–3 hours*	7 (9.0)
*> 3 hours*	49 (62.8)
Ever Received Training on Trans Women and HIV (*n* = 77)	
*Yes*	11 (14.3)
*No*	66 (85.7)

The vast majority of participants had received > 3 hours of specific training on the experiences/needs of people living with HIV (62.8%), with almost one-quarter (24.4%) reporting having received no prior training. Among those who provided qualitative responses, participants similarly received training from a province-wide LGBTQ training organization (*n* = 3), community-based organizations (*n* = 17), conferences/organized workshops (*n* = 9), workplace training programs (*n* = 3), and self-education (*n* = 2). Some indicated obtaining basic and advanced knowledge and care on treatment of HIV from an unspecified source (*n* = 16).

The majority of participants (85.7%) had never received training specific to the needs of trans women living with HIV. Among those who had and provided qualitative responses, some sources of training included a trans women HIV research initiative (*n* = 1), local community-based organizations (*n* = 1), and again self-education (*n* = 1).

### Intervention feasibility and acceptability

All 78 participants (100.0%) who began the intervention also consented to participate in the evaluation. Responses on pre- and post-intervention questionnaires were complete to different degrees (see Tables 2, 3, 4 and 5 for an indication of missing data). Pre- and post-intervention questionnaire completion rates were 65.4% (*n* = 51) for the gender-affirming HIV care measure and 71.2% (*n* = 56) for the gender-affirming healthcare measure. Participants took 15 to 20 min to complete pre- and post-intervention questionnaires, each.

Participants rated all aspects of the intervention highly, with facilitators rated the highest (Mean: 4.86 out of 5, SD: 0.39) and venue rated the lowest (Mean: 4.49, SD: 0.95) (Table 4). Participants attended for a variety of reasons, most notably the content (67.1%) and/or personal growth and development (73.4%). One-quarter (25.3%) attended at the requirement of their organization/manager. Almost all participants (91.9%) reported they would attend another training on trans women and HIV.

**Table 4:** Post-Intervention Feedback (*n* = 78)

Characteristic	Mean (SD) or N (%)
Rating on Workshop	
*Content (n = 76)*	4.53 (0.76)
*Facilitators (n = 71)*	4.86 (0.39)
*Registration Process (n = 73)*	4.80 (0.52)
*Venue (n = 78)*	4.49 (0.95)
Reason for Attending^a^	
*Content*	53 (67.1)
*Networking*	13 (16.5)
*Personal Growth and Development*	58 (73.4)
*Organization/manager Suggested Attendance*	23 (29.1)
*Organization/manager Required Attendance*	20 (25.3)
*Other*^*b*^	13 (16.5)
Attend Another Training on Trans Women and HIV (*n* = 74)	
*Yes*	68 (91.9)
*No*	6 (8.1)
*Maybe*	

^a^Categories not mutually exclusive, add up to > 100%

^b^Other reasons included (direct quotes of participants): Do better for my peers, for the better understanding on how to serve trans community members, I desire to learn to I can better help clients, Knowledge/improve my services, More trans knowledge, Part of our staff meeting, Peer navigators with PLWH including trans persons, The center did organization, To better understand and support my trans clients, To learn more, Training

Overall, 64 participants offered qualitative feedback about what was beneficial. Regarding content, participants described gender-affirming language, clarifications and explanations of new terms and concepts, the introduction of new theories and approaches, resources on health and rights of trans women living with HIV, and how and where to access treatment, care, and support as most beneficial (e.g., “*Learning about pronouns and the Ontario Human Rights Code as well as learning about the difference between sex, gender identity, expression, and sexual orientation*”; “*I learned about trans and HIV care and how to address people based on how they identify*”). Some learned about intersectionality for the first time, whereas others found the discussion of the concept “*an excellent reminder of the intersectionality of the experiences of trans feminine folk +HIV*.” Most beneficial aspects of delivery included the use of case studies, small groups, brainstorming, and discussion approaches for facilitation within a learning space (e.g., “*Learning and being in a space to ask questions in a respectful manner that was not judgmental*”; *“Great discussions from other providers/co-workers with diverse experiences*”).

Some gaps indicated by 46 participants included youth resources, and suggestions were made to provide more in-depth information about PrEP, post-exposure prophylaxis (PEP), hormone therapy and ART research evidence, as well as how barriers to HIV care, among trans women living with HIV, differ from those encountered among cis women also living with HIV. Other suggested areas for further explanation included mental health of trans women. Suggestions for improving the delivery centered on the inclusion of more stories of lived experiences from community partners either narratively or through videos (e.g., *“I think personal stories would have helped the understanding & reality of the situation….”*).

Overall feedback was overwhelmingly positive, with 24 participants of 38 providing overall feedback describing the intervention as either “*great*,” “*very informative*, *amazing facilitators*,” or an “*eye-opener*”. The most common critique was that the intervention could be more in-depth and better targeted towards participants’ prior knowledge and needs (e.g., *“I believe it would be helpful to have separate workshops based on the departments. Need & knowledge is different”*). Some participants who had prior training found sections on sex/gender and LGBTQ+ terms to be repetitive (e.g., *“Workshop should be for more targeted audience - content may not be new to many who have been working front line for trans”*).

### Perceived competency, attitudes/biases, and knowledge

Findings from the paired t-test analyses indicated statistically significant increases on each measure between pre- and post-intervention (Table 5). Knowledge, attitudes/biases, and perceived competency to provide gender-affirming HIV care improved by a mean difference (MD) of 8.49 (95% CI of MD: 6.12–10.85, *p* < 0.001, possible score range: 16–96) and knowledge, attitudes/biases, and perceived competency to provide gender-affirming healthcare by a MD of 3.21 (95% CI of MD: 1.90–4.90), *p* < 0.001, possible score range: 9–63) (Tables 4 and 5). Cohen’s d was 1.00 (strong) and 0.66 (moderate) for gender-affirming HIV care and gender-affirming care, respectively. While assumptions for parametric testing are met with sample sizes > 30, non-parametric testing (Wilcoxon signed rank tests) were conducted for a sensitivity analyses and also show significant differences from pre- to post-test for both outcomes at *p* < 0.001.

**Table 5:** Paired T-Tests to Assess Mean Differences in Pre- and Post-Intervention Measures

	Pre-Intervention		Post-Intervention				
Outcome	Mean	SD	Mean	SD	Mean Difference	95% CI of Mean Difference	*P* value
Gender-affirming HIV Care (*n* = 51)	75.57	12.96	84.06	8.60	8.49	6.12–10.86	< 0.001
Gender-affirming Healthcare (*n* = 56)	40.29	6.62	43.50	5.39	3.21	1.90–4.90	< 0.001

### Factors associated with pre- to post-intervention change scores

Bivariate analyses showed that number of trans clients served in the past year and number of transfeminine clients served in the past year were significantly negatively associated with pre- to post-intervention gender-affirming HIV care change score and gender-affirming care change score (r(51) = −0.297, *p <* 0.05 and r(51) = − 0.290, *p <* 0.05, respectively), suggesting that the higher the number of trans and transfeminine clients served in the past year, the smaller the change. Indirect service providers had a higher pre- to post-intervention gender-affirming HIV care change score compared to direct service providers (MD: 13.73, 95% CI of the MD: 1.5–25.9, *p <* 0.05). The number of past hours of training with trans communities was also significantly associated with pre- to post-intervention gender-affirming care change score (MD: 4.51, 95% CI of the MD: 1.96–7.07, *p <* 0.01), such that those with no past training had significantly higher mean change scores compared to those with any past training. The number of hours of past training about the needs/experiences of persons living with HIV was significantly associated with gender-affirming HIV care change score (MD: 7.60, 95% CI of MD: 1.98–13.22, *p <* 0.01), such that those with no past training had significantly higher mean change scores compared to those with any past training. Similarly, those who reported having not received previous training in trans women and HIV had significantly higher pre- to post-intervention gender-affirming HIV care change scores compared to those reporting any previous training (MD: 4.44, 95% CI of MD: 2.02–10.89, *p <* 0.05). This difference was also noted for the gender-affirming care change scores (MD: 3.58, 95% CI of MD: 1.14–6.00, *p <* 0.01).

## Discussion

This brief provider workshop pilot, TEACHH, showed promise for contributing to improved knowledge and perceived competency to provide gender-affirming HIV prevention and care among health and social service providers and providers-in-training, as well as high rates of feasibility and acceptability with most choosing to attend for personal/professional growth and indicating interest in further training on the topic. This pilot study also showed promise in improving gender-affirming provider knowledge, perceived competency, and attitudes/biases towards trans women living with and/or affected by HIV. This research joins a small number of studies that together demonstrate the effectiveness of trans competency and gender-affirmation training workshops [35, 36], and research evaluating provider interventions to reduce HIV stigma [40–44], demonstrating preliminary effectiveness of a pilot workshop that addresses the intersection of trans identity and HIV. The majority of participants had not received any prior training on the experiences of trans women and HIV prevention and care, suggesting TEACHH filled a critical gap at the nexus of gender-affirming care and HIV care. In this discussion, we reflect briefly on the content and delivery of TEACHH, making suggestions for further considerations relating to workshop development.

One question we posed in designing TEACHH was whether or not we could achieve a level of depth to address both HIV and trans-specific competency in a brief workshop, targeting providers at all levels of experience, across different types of care (e.g., medical, social), and making content applicable to those in both direct and indirect practice roles [57]. Interestingly, while there were some qualitative critiques of the level of content depth, when asked what the most beneficial aspect of the training was, many participants pointed out basic gender-affirming terminologies and language. We had a very diverse group of participants in terms of roles, with just over three-quarters holding direct service roles, with some in indirect roles (e.g., management). These findings suggest that TEACHH can address calls in both trans and HIV healthcare access literature to broaden training to include direct health and social service providers and administrative staff (e.g., front desk) [21, 42].

Participants also varied considerably with respect to past trans and HIV training experience. Not surprisingly, greater change in outcome measures from pre- to post-intervention was experienced by those with fewer trans and transfeminine clients served in the past year, those in indirect service roles, and those having received no prior training, suggesting that TEACHH may be particularly relevant for those with less trans and HIV experience. A future adaptation of TEACHH could consider a pre-group education session focused on foundational content (e.g., gender-affirming language) for those with no past trans training, allowing for greater opportunity to address intersectional considerations in a subsequent workshop and increase the benefits for those with prior training. Content specific to the needs and experiences of trans women living with HIV (e.g., health and rights of trans women living with HIV; how, where, and when to access HIV treatment, care and support) were noted as the most beneficial aspects of the workshop content, further underscoring the salience an intersectional approach [21, 22, 54–56]. Another option to address depth, may be to engage a physician who provides care to trans women (e.g., physician who prescribes both ART, PrEP and/or PEP and feminizing hormone therapy) to deliver part of the workshop.

That said, with respect to delivery, the quality of the facilitators (co-authors YP and KN) was rated higher than content, registration, and venue. YP and KN delivered trans-specific information trainings for more than a decade and 5 years, respectively, have long histories of HIV and trans advocacy/activism and research, including an emphasis on safety and rights of trans femme sex workers, were members of the Canadian HIV Trials Network (CTN) Trans People and HIV Working Group [69], and lead and coordinate several HIV and trans health research projects through the Trans Women HIV Research Initiative [70]. The facilitators’ extensive expertise on the topic areas and in delivering provider workshops resulted in the ability to support participants to work through case studies, group discussions, and to facilitate a non-judgmental space for personal and professional growth. While relatively little literature has examined how facilitator expertise and skillset influence participant outcomes with respect to provider interventions, a robust body of education literature has demonstrated that student achievement is influenced by years of teacher experience and teacher professional knowledge [71]. These findings suggest that future roll-out and scale-up of provider education interventions should utilized highly skilled facilitators.

Scant literature has compared the effectiveness of in-person versus virtual provider-level stigma-reduction interventions. However, a randomized controlled trial conducted with students showed effectiveness of a virtual intervention to reduce mental health stigma [72]. Another study comparing knowledge of evidence-based practices gained by providers attending in-person versus online delivery trainings showed equal gains across conditions [73]. Taken together and given the ongoing COVID-19 pandemic, these studies suggest a line of research assessing the effectiveness of TEACHH between virtual and in-person settings may be warranted. Virtual training, if effective, may increase scalability and reach of the TEACHH intervention to providers in suburban and rural settings.

One gap noted in the delivery was a need for more personal narratives throughout the pilot. As it is not always safe for facilitators with lived experience to share their own personal narratives while facilitating an intervention, despite evidence of the effectiveness of intergroup contact for promoting tolerant attitudes towards LGBTQ patients [33], future adaptations of TEACHH may make use of existing resources developed by members of the research team, generated through qualitative research, to share narratives of trans women living with and/or affected by HIV. For example, the team developed a series of open-access videos available on YouTube [74] on health and wellbeing of trans women, one of which addresses healthcare access and barriers [75].

Limitations include the pilot study design, which was not powered to address efficacy. The most notable limitation was the lack of a comparison group, which limited the ability to determine if changes observed were due to the workshop itself. Thus, our positive findings on outcome measures should be interpreted with caution. Moreover, the immediate pre- and post-workshop administration of measures precluded our ability to examine the long-term impacts. Other provider stigma reduction interventions with longer follow-up have found diminished effects over time [44]. For example, in their study assessing the efficacy of a participatory photovoice-informed provider HIV stigma intervention, Davtyan, Bartell, and Lakon [44] found that increased knowledge and improved attitudes towards people living with HIV at time two (one-week post intervention), had diminished at time three (three-months post intervention). A future randomized study design, using multiple timepoints of follow-up as has been carried out with HIV stigma provider interventions (e.g., Fernandez et al. [76]) could address these concerns. Moreover, future studies could assess the feasibility, needed frequency, and added benefits of refresher workshops to mitigate anti-trans and HIV stigma over time. Future studies may also strive for a more balanced sample in terms of medical versus social care providers by recruiting more physicians and nurses providing clinical care to rans women living with and/or affected by HIV.

Another way to strengthen the study design would be to measure the effects of TEACHH on behavioral intention and/or behavior change among providers. This could be done through provider observation during clinical interactions, as in a study by Varas-Díaz et al. [56]. Another option would be to examine the impact on clients/patients of those receiving the training, as was done in said study (Davtyan, Bartell, and Lakon [44]). Although changes in provider attitudes/biases were observed at time one, investigators found that the training did not have an impact on observations PLWH of enacted stigma at either time one or time two follow-up [44], further underscoring the importance of examining the impact on patients themselves. While the strong and moderate effect sizes noted for the change in gender-affirming HIV care and gender-affirming care, respectively, are promising, in the absence of patient-level data it is difficult to interpret clinical significance. All study measures were self-reported and therefore may be impacted by social desirability bias. However, the newly developed measures, particularly regarding gender-affirming HIV care, had high and adequate reliability. As we did not ask participants about the feasibility of integrating TEACHH on larger scale within their organizations, future research may also seek to identify barriers (e.g., time/availability of service providers) and facilitators (e.g., support and attendance of management) to scale-up of TEACHH. Moreover, future research could examine ways to increase completion rates of pre- and post-intervention questionnaires. Notably, the main strength of our study was the community-based approach [58] and, specifically, the conceptualization of the workshop by the extensive experience of team co-investigators YP, KN, and GL, and formative focus groups with trans women and service providers [22].

Provider interventions targeting those already in practice are not sufficient to shift the organizational culture, knowledge and competency about the needs/experiences of trans women and HIV prevention and care. While one component of the TEACHH intervention provided information on strategies for enhancing gender-affirming service provision at the organizational level, we did not assess organizational-level outcomes. Future adaptations of TEACHH could draw on the robust body of literature focused on how to create affirming clinic environments (e.g, [77, 78]) and measure organizational-level changes related to the physical clinic space (e.g., trans-inclusive posters/artwork/magazines, gender-inclusive bathrooms or a gender-inclusive bathroom policy, inclusive intake forms).

Notably, only four participants indicated having received any trans-specific training in a formal college/university-level course. This is consistent with a plethora of research documenting low hours of LGBTQ content in medical and allied health professional training [26, 27]. Other studies have highlighted how medical power and authority sustain social and institutional stigma against trans people by excluding knowledge of their care from medical training [79], evidenced in the limited hours dedicated in medical schools to lesbian, gay, bisexual, trans, and queer (LGBTQ+) health issues [80]. Thus, to shift healthcare practices, such training should be incorporated in allied health professional education at a college and university levels and residency programs [81, 82] with a higher number of hours [27].

Additionally, allied healthcare professional education could take a regional approach and seek to foster competent and affirming care in rural settings [83]. For example, in their LGBTQ curriculum pathway, the University of Washington School of Medicine required students to engage in advocacy/community activities with rural LGBTQ+ organizations [83]. Indeed, while TEACHH took place in urban settings, rural care of trans women and trans women living with/affected by HIV is of critical importance, given studies showing perceived inadequacies in rural provider competency to provide affirming care and subsequent challenges faced by rural LGBTQ people, including higher rates of discrimination, lower likelihood of sexual/gender minority identity disclosure, and health disparities [84]. A tailored approached to adapting TEACHH could involve engaging with local LGBTQ community members/leaders, building relationships between community members/leaders and local health care organizations, and engaging community members/leaders in adaptation and delivery of TEACHH [85]. An overall structural level intervention could include hiring trans women as independent researchers, as health professionals who lead and contribute to interdisciplinary medical teams, and/or as peer educators and researchers [17, 47]. Where implicit motivation to attend trainings and/or provide affirming care are lacking, legal human rights protections may be leveraged to enforce a standard of care to which trans women are entitled [86].

## Conclusions

Trans women face many barriers to accessing HIV prevention and care, notably lack of knowledge and competency as well as trans-related and HIV-related stigma among their providers [16–22]. The TEACHH pilot – a theoretically-informed, brief workshop – shows promise for addressing these critical gaps. This pilot study suggests scale-up and widespread implementation of TEACHH may increase access to gender-affirming HIV prevention and care for trans women, ultimately reducing health disparities.

## Data Availability

The dataset generated and analysed during the current study is not publicly available due to confidentiality agreements with the University of Toronto but are available from the corresponding author (lacombed@umich.edu) upon reasonable request.
